# Probiotics: Health Benefits in Relation to Gut Microbiota and Functional Food Applications

**DOI:** 10.3390/foods15112004

**Published:** 2026-06-04

**Authors:** Yi Xu

**Affiliations:** School of Food and Biological Engineering, Hefei University of Technology, Hefei 230009, China; xuyixuyi3734@163.com

## 1. Introduction

Probiotics, as ratified by the International Scientific Association for Probiotics and Prebiotics (ISAPP) consensus that endorsed and refined the original FAO/WHO definition, are “live micro-organisms that, when administered in adequate amounts, confer a health benefit on the host” [1,2]. Over the past two decades, this concept has evolved from a niche category of dietary supplements into an essential part of contemporary nutritional science. The mounting attention has grown in parallel with our deepening appreciation of the gut microbiota as a critical regulator of host physiology, which impacts digestion, modulates immunity, calibrates energy metabolism, and, through the microbiota–gut–brain axis (MGBA), reaches organs as remote as the central nervous system [3,4,5]. Concomitantly, the definition of probiotics has been progressively augmented by prebiotics, symbiotics, and postbiotics (preparations of inanimate micro-organisms and/or their components that confer a health benefit) [6,7]. This development has, in turn, transformed the landscape of functional food research: food matrices are attracting greater appreciation for their ability to deliver and sustain the probiotic properties of the accountable microbial strains.

Despite this broad consensus on probiotics and health, the field still grapples with two enduring questions. Firstly, how do specific probiotic strains exert their benefits at the systemic, cellular, and molecular levels? This question should be addressed in contextualized settings, which should specify the species or even strain-level identification of probiotics, the physiological background with possible relevance to disease, or gut microbiota status. Secondly, how can these mechanistic insights be translated into food formulations and dietary regimens that retain efficacy outside the laboratory? This has substantial relevance to the specific translational validity from animal experiments to population-based trials. Here, food is conceptualized broadly: it spans ingredients, prototype products, market-ready formulations, dishes, and even whole diets.

To this end, this Special Issue of *Foods*, “Probiotics: Health Benefits in Relation to Gut Microbiota and Functional Food Applications,” aimed to gather cutting-edge research addressing the above questions. After a careful peer review process, eight contributions were accepted, encompassing one randomized human clinical trial, three mechanistic animal studies, two food matrix or fermentation-based investigations, and two reviews. The aim of the Special Issue—to explore how probiotics interface with the host across diverse physiological contexts and food formats—is therefore well represented in this collection.

## 2. Overview of the Published Articles

To assist readers in navigating this collection, we have selected five representative papers to illustrate key findings and contributions to probiotic research.

The first contribution by Sun et al. reports a randomized, double-blind, placebo-controlled clinical trial that evaluated the Wilac L probiotic complex—a combination of *Levilactobacillus brevis* WiKim0168 and *Leuconostoc mesenteroides* WiKim0172 isolated from kimchi—for its capacity to ameliorate alcohol-related hangovers (Contribution 1). Twenty-six healthy participants were randomized into six test sequences, with crossover after an interval of 7–10 days. Notably, blood acetaldehyde concentrations after Wilac L administration were significantly lower than those of the control group, while subjective hangover symptoms were correspondingly mitigated and no adverse events were recorded. This study provides an instructive example of how strains derived from a traditional fermented food can be repurposed into functional ingredients that target an everyday health concern. This finding is in line with a recent investigation which discovered that the probiotic VITA-PB2 effectively alleviates alcohol consumption and hangover symptoms in 28 healthy adults [8].

Santos et al. provided a comprehensive review centered on probiotic and “smart probiotic” micro-organisms in the management of inflammatory bowel diseases (IBDs), considered specifically as live biotherapeutics interfacing with the food matrix (Contribution 2). The authors curated the literature retrieved from large-scale databases up to October 2024. While conventional probiotic strains are not yet enshrined in standard IBD treatment protocols, the authors make a persuasive argument that the convergence of biotechnology and nutrition represents a credible therapeutic horizon. The review is most useful in filling the standardization gaps that currently constrain clinical translation, thereby outlining a roadmap that benefits both research scientists and food industry participants.

Zhu et al. investigates whether royal jelly can transfer its caste-modulatory ability from honeybees to mammals through a specific microbiota–gut–brain axis (Contribution 3). Using a vancomycin-induced dysbiosis paradigm in Sprague Dawley rats, the authors found that two weeks of RJ supplementation (2.5 g/kg, postnatal weeks 6–7) significantly restored the social rank of submissive rats, as evidenced by agonistic, water competition, and tube tests. Specifically, the intestinal abundance of *Proteobacteria*, particularly *Escherichia* and *Clostridium*, was lowered to a level comparable with that of dominant rats, which was indicative of a potential negative correlation. Transcriptomic profiling of the medial prefrontal cortex revealed coordinated remodeling of pathways involved in neuronal transmission, with gastrin-releasing peptide (*Grp*/*Grpr*) signaling emerging as a candidate axis bridging the gut and the brain. The functional necessity of this axis was further validated by gavage with *Escherichia*, which reverted RJ-treated rats to a submissive state and concordantly altered cerebral genes such as *Grpr* and *Grpel1*. By integrating microbiome, transcriptome, and behavioral data, this study sheds light on the mechanistic insight through which a traditional functional food can rebalance the gut–brain crosstalk in higher organisms.

Wang et al. addresses an often-overlooked dimension of probiotic efficacy: the food matrix in which the probiotic is delivered and the timing at which it is consumed (Contribution 4). Through an in vitro simulation of human gastrointestinal digestion, the authors quantified the survival of *Lactobacillus rhamnosus* GG (LGG), a well-defined probiotic strain, when co-ingested with durum wheat pasta or soy milk at three timepoints (on an empty stomach, with a meal, and after a meal). According to the data, pasta outperforms soy milk in protecting LGG, with viable counts of 5.92–6.38 versus 4.93–5.39 log CFU/g. Co-ingestion or post-meal administration further augmented the bacterial viability in the studied context. Likewise, LGG co-ingestion also facilitated starch and protein digestion, increasing starch digestibility from 84.80% to 89.00% and soymilk protein digestibility from 78.00% to 80.00%, suggesting a synergistic interaction between the probiotic and the food matrix. Taken together, this study fills a gap in mixed use of dietary regime and probiotics to reach an optimal efficacy, which underscores the importance in formulated products in functional food development.

Another contribution by Bogdanović et al. expands this Special Issue into the territory of sustainable food biotechnology (Contribution 5). Faced with the approximately 30 million tons of byproducts generated annually by the medicinal plants industry, the authors propose the fermentation of common nettle (*Urtica dioica* L.) tea byproducts and flowers with *Ligilactobacillus salivarius* ATCC 11741 as a green valorization strategy. Lyophilized aqueous and ethanolic extracts were subjected to fermentation, and the process simultaneously increased phenolic content and antioxidant activity while preserving probiotic viability, thereby producing a dual perspective: a bioactive-enriched extract and a viable probiotic biomass. This contribution illustrates how probiotic technology can serve as the bridge between functional food development and the circular utilization, and reinforces the broader thesis that probiotic, prebiotic, and prebiotic-like ingredients can be co-engineered within a single food matrix [6].

## 3. Cross-Cutting Reflections

Beyond the individual contributions, this Special Issue invites a broader stocktaking. In what follows, four cross-cutting threads are woven together: the breadth of disease targets that probiotics now address, the relationship between probiotics and fecal microbiota transplantation (FMT), the rising profile of postbiotics, and the role of *Bifidobacterium* and other autochthonous microbes as anchors of gut microbiota eubiosis.

### 3.1. Probiotic Interventions Across Diverse Diseases: Association and Dissociation

The eight contributions of this Special Issue alone testify to the remarkable breadth of conditions for which probiotics are currently being investigated. Within a single collection, we encounter probiotic interventions targeting alcohol-induced acetaldehyde accumulation (Contribution 1), inflammatory bowel diseases (Contribution 2), exercise-induced fatigue (Contribution 6), social rank deficits arising from gut dysbiosis (Contribution 3), colorectal carcinogenesis (Contribution 7). The list does not exhaust the spectrum: recent randomized controlled trials have added migraine headaches to the probiotic agenda, with twelve weeks of probiotic and vitamin D co-supplementation yielding significant reductions in attack frequency and severity [9]; comprehensive reviews continue to revisit the brain–gut axis in IBD and other functional gastrointestinal disorders, where psychological comorbidities are seen as integral, rather than incidental, dimensions of disease activity [10].

What is striking, however, is the apparent lack of an obvious common thread among such disparate targets—a duality that may be summarized as the “association–dissociation” nature of probiotic research. On the one hand, every one of these conditions is associated, in one way or another, with documented alterations of the gut microbiota, suggesting that probiotic interventions genuinely converge on a shared mechanistic substrate. The MGBA, the gut–immune axis, and microbe-derived metabolites collectively provide a small set of universal channels through which probiotics can influence remote organs, irrespective of the surface diagnosis. On the other hand, the specific microbe–disease pairings, dose–response relationships, and effect sizes diverge sharply across pathologies, and the evidence base is conspicuously uneven: clinical-grade evidence is robust for a handful of conditions (acute infectious diarrhea, antibiotic-associated diarrhea, and irritable bowel syndrome), while remaining preliminary for others (migraine, social hierarchy, and colorectal cancer prevention). This dissociation between mechanistic plausibility and disease-level certainty is, in many respects, the central source of tension in the field [1,7]. Resolving it will require strain-level clarity, mechanism-anchored hypotheses, and adequately powered population-level trials—exactly the prescription advanced, in different ways, by every contribution of this Special Issue.

### 3.2. Probiotics, FMT, and the Postbiotic Frontier

Among microbiota-based interventions, probiotics now sit at one end of a continuum that stretches to fecal microbiota transplantation (FMT) at the other extreme. As discussed in Contribution 7, FMT has emerged as a viable adjunct in oncological settings and as a treatment of considerable promise for recurrent *Clostridioides difficile* infection, owing to its capacity to completely rewrite the recipient’s intestinal community. Probiotic supplementation, by contrast, offers a more controlled, regulator-friendly modality: defined strains, defined doses, and defined safety profiles, with the predictability that is essential for food and supplement applications. Despite differing in scale and complexity, the two strategies share a central goal—the restoration of microbial eubiosis—and draw on overlapping mechanistic concepts. The convergence is most evident in their efficacy signatures: both modalities tend to attenuate dysbiosis-associated taxa, expand short-chain fatty acid producers, and re-establish microbial diversity, regardless of the entry-point disease. It is therefore unsurprising to see hybrid strategies—synbiotics, defined bacterial consortia, and complementary FMT-plus-probiotic regimens—gaining traction in the literature.

A further conceptual extension is offered by postbiotics: preparations of inanimate micro-organisms and/or their components, together with their metabolites, that confer a health benefit on the host [5,7]. Postbiotics inherit the regulatory and supply-chain elegance of conventional functional food ingredients while sidestepping the fragility of live cells, with corresponding advantages in shelf life, dose precision, and safety in vulnerable populations such as infants, the elderly, and the immunocompromised. Several findings reported in the present collection implicitly point toward the postbiotic concept: the short-chain fatty acid signatures associated with *Weizmannia coagulans* BC99 (Contribution 6), the metabolic remodeling driven by *Clostridium butyricum* WL-53 (Contribution 8), and the polyphenol enrichment achieved by *Ligilactobacillus salivarius* fermentation of nettle (Contribution 5) all suggest that a meaningful portion of the observed benefit may be attributable not to the live cells themselves but to the bacterial metabolites and structural components they leave behind. Future studies that explicitly compare matched live-cell, heat-inactivated, and metabolite-only preparations within a single experimental paradigm will be valuable for demarcating the live-versus-postbiotic contributions to functional food efficacy.

### 3.3. Bifidobacterium and Autochthonous Microbes as Anchors of Eubiosis

If a single genus can be said to embody the concept of gut-microbiota eubiosis in modern probiotic research, it is *Bifidobacterium*. Members of this genus are among the earliest microbial colonizers of the newborn intestine, can constitute over 90% of the bacterial population in breast-fed infants, and persist throughout life as influential, if quantitatively minor, members of the adult colon [11]. As autochthonous (native-niche) inhabitants, bifidobacteria participate in immune-system maturation, fermentation of human milk oligosaccharides and other complex carbohydrates, and the production of short-chain fatty acids that nourish enterocytes and signal to the host. Many of the most popular probiotic preparations are deliberately built around *Bifidobacterium* species—alone or paired with *Lactobacillus* sensu lato—precisely because their re-introduction tends to restore, rather than perturb, the indigenous community structure. This is the operative distinction between probiotic supplementation and microbial colonization in the strict ecological sense: the most desirable probiotic strains behave less like invaders and more like reinforcements of an existing equilibrium. 

This privileged status of native-niche genera is borne out, even if not always foregrounded, in the contributions of this Special Issue. Contribution 8 reports that *Clostridium butyricum* WL-53 supplementation enriches “beneficial genera” while attenuating the over-representation of certain *Firmicutes* in HFD-fed mice; Contribution 6 documents the augmentation of short-chain fatty acid producers, including *Roseburia*, *Mucispirillum*, *Rikenella*, and *Kineothrix*; Contribution 3 shows that the recovery of dominance behavior is paralleled by the attenuation of *Proteobacteria*, *Escherichia*, and *Clostridium* overgrowth toward a profile resembling that of healthy controls; and Contribution 5 demonstrates that fermentation by *Ligilactobacillus salivarius* can simultaneously deliver bioactive metabolites and a viable probiotic biomass. Across these distinct settings, the recurring metric of success is the same: a measurable rebalancing of the microbial community toward the kind of profile observed in healthy hosts, with bifidobacteria and other autochthonous taxa frequently emerging as informative indicators. The implication for functional food development is straightforward—probiotic interventions are most likely to deliver durable benefits when they reinforce, rather than displace, the indigenous microbiota, and when their evaluation includes eubiosis metrics alongside the traditional clinical endpoints.

## 4. Conclusions

When viewed collectively, the contributions of this Special Issue trace a coherent trajectory within the field of probiotic research. A few thematic threads run through this collection. First, the gut microbiota emerges as the central pivot through which probiotics achieve their effects with regard to numerous symptoms or physiologies such as hangover relief, colon cancer migration or social dominance. Gut microbiota primarily act by modulating the MGB axis, which exerts impact on distant organs like the central nervous system, in which microbiota-derived metabolites play essential roles by interacting with other molecules. Second, multi-omics profiling, metabolomic mapping, and transcriptomic analysis are becoming baseline expectations for high-quality probiotic research, and have proven particularly adept at identifying novel molecular targets and providing clues to elicit reciprocal actions. Third, food matrices and processing exert a major influence on probiotic performance, suggesting that the effect of a probiotic strain cannot be evaluated independently from the food in which it is delivered. In future perspectives, more attention should be garnered from the associations and dissociations of diseases that are intervenable by probiotics, postbiotic frontiers and roles of bifidobacteria as an eubiosis balancer for autochthonous microbes.

I hope that the readers of *Foods* will find both useful technical insights and a compelling argument for cross-disciplinary thinking in this Special Issue. As Guest Editor, I would like to encourage the publication of further studies that bridge microbiological detail and food engineering pragmatism. The growing body of work gives us a reason to believe that the next generation of functional foods will be rationally designed to integrate live probiotics, postbiotic preparations, food matrix and FMT-derived insights into a composite, rather than comprising a simple combination of micro-organisms and ingredients.
